# Acute MRSA Sinusitis with Intracranial Extension and Marginal Vancomycin Susceptibility

**DOI:** 10.1155/2013/153239

**Published:** 2013-09-11

**Authors:** Parvathi S. Kumar, Kenji M. Cunnion

**Affiliations:** Department of Pediatrics, Division of Infectious Diseases, Children's Hospital of the King's Daughters and EVMS, Norfolk, VA 23507, USA

## Abstract

Methicillin resistant *Staphylococcus aureus* (MRSA) is increasingly being described as a cause of acute sinusitis. We present a patient with acute MRSA sinusitis complicated by rapid intracranial extension, marginal vancomycin susceptibility (MIC = 2 mg/L), delayed drainage of intracranial abscess, and subsequent development of rifampin resistance. Given the relatively high risk of intracranial extension of severe acute bacterial sinusitis and high mortality associated with invasive MRSA infections, we suggest early surgical drainage of intracranial abscesses in these circumstances. We believe this is important given the limited intracranial penetration of currently available treatment options for MRSA, especially those with a vancomycin minimal inhibitory concentration (MIC) of ≥2 mg/L.

## 1. Introduction

Our patient, a previously healthy 12-year-old male, presented with acute MRSA sinusitis and rapid intracranial extension. The clinical case was complicated by a marginal vancomycin susceptibility (MIC = 2 mg/L), delayed drainage of intracranial abscess, and development of rifampin resistance. This case is illustrative of community-associated MRSA as a potential cause of acute sinusitis leading to intracranial extension, the challenges of antibiotic management of intracranial MRSA abscess, and the hazards of delayed drainage of intracranial MRSA abscess.

## 2. Case Presentation

A previously healthy 12-year-old male with a history of intermittent migraines was admitted with acute onset of altered mental status and facial swelling. The patient had symptoms of headache, “upset stomach,” increasing fatigue, and tactile fever for two days prior to admission. On the day of admission he was found to be minimally responsive with significant swelling to the left aspect of the face, yellowish discharge from the left eye, and a protuberance from the forehead. A noncontrast head CT scan done at an outside health care facility demonstrated bilateral orbital cellulitis, pansinusitis, and possible venous sinus thrombosis prompting transfer to a pediatric hospital.

**Figure 1:**
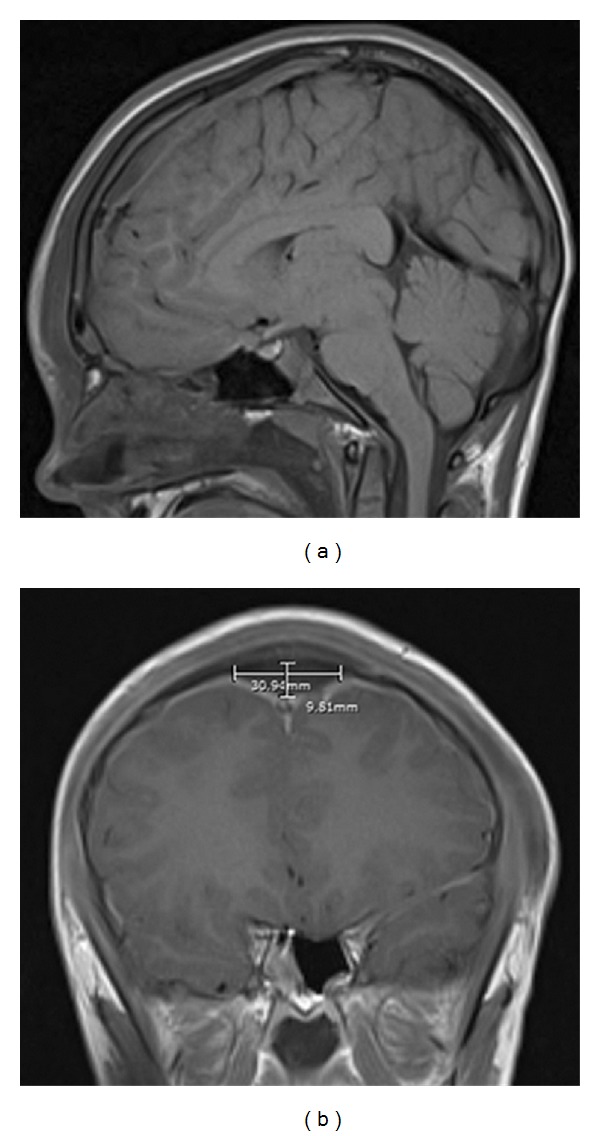
(a) MRI of the brain showing sagittal T1-weighted image after administration of contrast. The fluid collection reported to have a thin enhancing wall ran along the dorsal aspect of the superior sagittal sinus. (b) MRI of the brain showing coronal T1-weighted image after administration of contrast. Diffuse smooth dural enhancement is noted, bilaterally.

On admission, the complete blood count (CBC) revealed a white blood cell count of 8400 cells/*μ*L with a manual differential of 8% bands, 3% metamyelocytes, 74% neutrophils and 9% lymphocytes, hemoglobin (gm/dL)/hematocrit (%) of 8.2/24.3, and platelet count of 101 × 10^3^/*μ*L. Additionally, a coagulation panel revealed a prolonged prothrombin time (PT) and activated partial thromboplastin time (aPTT) of 19 seconds and 42.3 seconds, respectively, as well elevated D-dimer levels of 11.11 mg/L suggestive of disseminated intravascular coagulation in this patient. A brain MRI showed a superior midline epidural fluid collection, measuring 8.6 cm anteroposterior × 3.1 cm transverse × 1.0 cm craniocaudal, following the dorsal aspect of the superior sagittal sinus (Figures 1(a) and 1(b)). The fluid collection demonstrated a thin enhancing wall, and diffuse smooth dural enhancement was noted bilaterally. Additionally, a tiny focus of intracranial air was present posterior and superior to the opacified frontal sinuses. Pansinusitis with bilateral orbital cellulitis was also reported. An MRA/MRV of the head noted mild narrowing of the anterior sagittal sinus by the adjacent epidural abscess, but no occlusion of the sinus and no evidence of thrombosis. He was intubated and sedated due to severely depressed mentation. Physical examination was notable for a temperature of 39.0°C with diffuse facial swelling, protuberant eyelids, yellow discharge from both nares, and a mildly protuberant soft mass in the lower midline forehead consistent with Pott's puffy tumor. Antibiotics were initiated with cefotaxime, vancomycin, and metronidazole.

Neurosurgical consultation was requested for surgical drainage of the intracranial abscess, but operative intervention was refused citing that the collection was epidural and not contributing to a mass effect. Neurosurgical consultants questioned whether the collection was an abscess, despite the patient's history, symptomatology, and imaging strongly suggesting that this was a case of acute bacterial sinusitis with rapid extension intracranially, orbitally, and subcutaneously. 

Initial peripheral blood culture was positive at 14 hours of incubation and identified as MRSA. Significant concern was raised about the MRSA having an MIC of 2 mg/L, which is the upper limit of susceptible, given the challenges of vancomycin penetration into an intracranial abscess. Because the epidural MRSA abscess was not drained initially, rifampin was added. It took 4 days to achieve a target vancomycin trough level of 17 *μ*g/mL. 

The patient remained febrile, clinically unstable, and intubated during the first three days of hospitalization. A repeat MRI on day three of hospitalization was interpreted by the radiologist as a mild increase in size of the epidural abscess in the superior midline, but surgical intervention was again deferred by neurosurgical consultants. The patient's clinical status did not improve on antibiotics, and a head MRI on the sixth day of hospitalization showed an increased size of the epidural hematoma with a new abscess along the outer table of the left frontal bone and a small subdural fluid collection along the anterior left frontal lobe. A craniotomy procedure with abscess drainage was then performed recovering purulent fluid, all cultures of which grew MRSA, demonstrating the persistence of the organism in the intracranial abscess. On hospital day ten otolaryngology surgeons performed bilateral maxillary antrostomies, bilateral ethmoidectomies, bilateral frontal sinus drainage, and left orbital subperiosteal abscess drainage. MRSA recovered from the frontal sinus demonstrated new resistance to rifampin, which was then discontinued. On hospital day thirteen a right-sided thoracotomy was performed to drain an empyema, which grew MRSA. After four weeks of hospitalization the patient was discharged home on antibiotics and has subsequently made a complete recovery. Although a formal evaluation of the patient's immune system was not performed, he had a normal globulin fraction of 3 gm/dL suggesting that a major deficiency of antibody production was unlikely.

## 3. Discussion

Community-associated MRSA has been the predominant cause of skin and soft tissue infections in North America for the past decade [1, 2]. *Staphylococcus aureus* has long been appreciated as a causative agent of chronic bacterial sinusitis [3], but MRSA as a cause of acute maxillary and sphenoid sinusitis has been only recently appreciated [4, 5]. To our knowledge this is the first report of an immunocompetent child with acute MRSA sinusitis complicated by rapid intracranial extension. It is reasonable to expect that this scenario will occur more frequently in the future given that the reported risk of intracranial extension in patients hospitalized with a diagnosis of sinusitis ranges from 3.7 to 11% [6, 7]. Recognition of the possibility of acute MRSA sinusitis leading to intracranial infection is vital due to the high risk of morbidity and mortality associated with either intracranial complications of acute sinusitis of 5–10% [8] or invasive MRSA infections of 17–32% [9–11]. Given that antibiotic treatment of acute sinusitis with intracranial extension is unlikely to be initially directed against MRSA, the risk of mortality may be even higher for MRSA sinusitis with intracranial extension. This scenario also emphasizes the importance of achieving a microbiological diagnosis or risk delaying appropriate antibiotic management.

This case additionally illustrates the importance of timely drainage of an intracranial MRSA abscess. MRSA subperiosteal abscesses in orbital infections have been noted to be increasing in incidence and are associate with a more aggressive disease course than for other organisms, leading to recommendations for empiric antibiotic coverage with a very low threshold for surgical intervention [12–15]. It is reasonable that similar recommendations for timely surgical management be applied in the setting of MRSA sinusitis with intracranial extensions, given limited CNS penetration for vancomycin of 7–14% of serum concentration [16]. Limited vancomycin penetration into abscesses [17] additionally compromises antimicrobial effectiveness. These challenges are especially daunting in the face of MRSA with a marginal vancomycin MIC of 2 mg/L, which has been frequently associated with antibiotic failure in a variety of clinical settings [18, 19]. For our patient, six days of vancomycin therapy did not sterilize or prevent extension of the abscess, emphasizing the importance of timely surgical debridement. Gallagher et al. concluded that optimal treatment of suppurative intracranial complications of sinusitis is debridement of the paranasal sinuses in combination with neurosurgical drainage of the intracranial focus and intravenous antibiotics [20].

Delayed surgical intervention has also been associated with increasing MIC values for vancomycin leading to the development of resistance (VISA) and heteroresistance (hVISA) [21]. Although increasing vancomycin MIC did not occur for this patient, his MRSA did develop rifampin resistance while on vancomycin and rifampin. This likely represented inadequate vancomycin antimicrobial activity in purulent fluid collections consistent with delayed surgical debridement and the marginal vancomycin MIC for this MRSA.
